# Prevalence and factors associated with hematological adverse events in RR-TB patients on linezolid-based regimens in Uganda: a multicenter retrospective cohort study

**DOI:** 10.1186/s12879-026-13405-4

**Published:** 2026-04-30

**Authors:** Esther Apio, Joseph Baruch Baluku, Denis Bwayo, John Peter Masaba, Erias Kirabo, Jasper Kebesu, Oreb Nankunda, Julius Imalingat, Richard Katuramu

**Affiliations:** 1 Department of Internal Medicine, Busitema University, Tororo, Uganda; 2 Department of Internal Medicine, Mbale Regional Referral Hospital, PO Box 1460, Mbale, Uganda; 3 Division of Pulmonology, Kiruddu National Referral Hospital, Kampala, Uganda

**Keywords:** Linezolid, Rifampicin-resistant tuberculosis, Hematological AEs, Multicenter retrospective cohort study, Uganda

## Abstract

**Background:**

Linezolid (LZD) is a cornerstone of rifampicin-resistant tuberculosis (RR-TB) treatment, yet its hematological adverse events (AEs), particularly anemia, leukopenia, and thrombocytopenia, remain understudied in resource-limited settings like Uganda. With a high RR-TB burden and comorbidities such as HIV and malnutrition, understanding the prevalence and risk factors of these hematological AEs is critical to improving treatment adherence and outcomes.

**Aim:**

This study aimed to determine the prevalence of hematological AEs and identify factors associated with hematological AEs among RR-TB patients receiving LZD-based regimens in Uganda.

**Methodology:**

A multicenter retrospective cohort study was conducted using medical records of RR-TB patients treated with LZD at four Ugandan hospitals (Mbale RRH, Iganga General Hospital, Moroto RRH, and St. Kizito Matany Hospital) from 2020 to 2024. Hematological AEs were defined as anemia (Hb < 12 g/dL in women or < 13 g/dL in men), thrombocytopenia (platelets < 150 × 10³/µL), and leukopenia (WBC < 3.7 × 10⁹/L). Sociodemographic, clinical, and treatment-related data were extracted. A modified Poisson regression model with robust standard errors was used to estimate adjusted prevalence ratios (aPR) and 95% confidence intervals (CI).

**Results:**

Among 412 patients, 62.9% (259/412) had at least one hematological AE at baseline or during follow-up, and 36.4% (150/412) had hematological AEs first detected after LZD initiation. Among patients with available baseline CBC, 40.0% (98/245) developed incident hematological AEs. First detected anemia, thrombocytopenia, and leukopenia occurred in 21.4% (88/412), 14.8% (61/412), and 13.8% (57/412) of patients, respectively. Rural residence (aPR 1.60, 95% CI 1.11–2.33) and divorced or widowed marital status (aPR 4.10, 95% CI 1.58–10.77) were independently associated with hematological AEs. Patients with hematological AEs had lower treatment success (83.0% vs. 93.5%) and higher loss to follow-up (15.1% vs. 5.9%) than those without AEs.

**Conclusion:**

Hematological AEs were frequent among RR-TB patients receiving LZD in Uganda and were associated with reduced treatment success and increased loss to follow-up, while mortality remained low. Rural residence and divorced or widowed marital status were independently associated with these events, highlighting the need for targeted monitoring in higher-risk groups.

**Clinical trial number:**

Not applicable.

**Supplementary Information:**

The online version contains supplementary material available at 10.1186/s12879-026-13405-4.

## Introduction

Drug-resistant tuberculosis (DR-TB) is caused by Mycobacterium tuberculosis strains resistant to the most effective anti-TB medicines. Multidrug-resistant tuberculosis (MDR-TB) involves resistance to both isoniazid and rifampicin, while rifampicin-resistant TB (RR-TB) includes resistance to rifampicin with or without other drugs [1, 2]. Together, MDR/RR-TB poses a major global health threat. In 2023, an estimated 400,000 people developed RR-TB, and the treatment success rate (TSR) remained at only 68%, much lower than outcomes for drug-susceptible TB [3].

In Sub-Saharan Africa, MDR and RR-TB outcomes remain poor, with TSR ranging between 55% and 65%, and high loss to follow-up and mortality [4, 5]. Linezolid (LZD)-related hematological complications have also been widely reported in this region. In South Africa, Letswee et al. (2019) documented anemia and thrombocytopenia as common AEs that often required dose reduction or discontinuation [6]. In Nigeria, Dayyab et al. (2021) found that 14% of patients discontinued treatment because of severe adverse effects, including hematological disorders [5]. These findings highlight that hematological AEs are a major barrier to successful MDR/RR-TB treatment in African cohorts.

Uganda is among the 30 high-TB/HIV burden countries [7]. In 2024, approximately 99,000 people developed TB, with nearly 1,900 new MDR/RR-TB cases recorded [8, 9]. However, 807 people with MDR/RR-TB were started on treatment in 2024, indicating a persistent gap between incidence and treatment initiation [8]. Limited access to rapid molecular diagnostics remains a key barrier to timely case detection, with 72% of new bacteriologically confirmed pulmonary TB patients tested for rifampicin resistance. The treatment success rate (TSR) for MDR/RR-TB in Uganda has improved to 89% (based on the 2022 cohort), though mortality and loss to follow-up persist as programmatic challenges [8, 9].

Treatment of RR-TB is complex and lengthy, typically lasting 9–24 months and requiring multiple drugs. Until recently, regimens included injectable agents with long treatment durations and substantial AEs. In 2019, the WHO revised its guidelines to recommend all-oral regimens that prioritize Group A drugs, including bedaquiline, fluoroquinolones, and LZD [10, 11]. The introduction of these regimens has improved outcomes globally, but adverse drug reactions remain an important cause of treatment interruption. Recent trials, including the endTB trial, have further demonstrated the efficacy and safety of all-oral LZD-containing regimens for RR-TB [12].

LZD is a key Group A drug and is widely used in both short and long RR-TB regimens. While it is associated with improved treatment outcomes, its use is limited by adverse effects such as lactic acidosis, peripheral neuropathy, optic neuropathy, and hematological AEs [11, 13].

These AEs often necessitate dose reduction or discontinuation, which can compromise treatment efficacy [14].

However, data on the prevalence and risk factors for LZD-associated hematological AEs from Uganda and similar low-resource, high HIV-burden settings are scarce. Understanding the local epidemiology, including risk factors and timing of hematological AEs, is essential for optimizing monitoring strategies, preventing treatment interruptions, and improving outcomes in this high-burden setting.

## Methods

### Study design and setting

A multicenter retrospective cohort study was conducted using patient medical records from January 2020 to December 2024 across four hospitals in Uganda: Mbale Regional Referral Hospital, Moroto Regional Referral Hospital, Iganga General Hospital, and St. Kizito Matany Hospital.

### Sample size estimation

Sample size was estimated using OpenEpi version 3.01, assuming a two-sided 95% confidence level, 80% power, and a 1:1 unexposed-to-exposed ratio. Based on a prior study reporting a 46% prevalence of hematological AEs with LZD outcome risks, they were set at 31% and 46% [15]. The minimum required sample size was 328, and 412 participants were included to account for incomplete records.

### Study population and data collection

The study population comprised adults (≥ 18 years) with microbiologically confirmed RR-TB who initiated an LZD-containing regimen within the study period. Eligibility required confirmation of RR-TB by Xpert MTB/RIF, Line Probe Assay (LPA), or culture; receipt of LZD for at least one week; and availability of at least one complete blood count (CBC) result after LZD initiation. Patients with documented pre-existing hematological disorders were excluded. Data on demographics, clinical characteristics, serial laboratory measurements (including CBCs), and final treatment outcomes were abstracted from medical records using a standardized electronic form. For patients with available pre-treatment laboratory data, baseline CBCs were used to define and analyze strictly incident hematological AEs.

All participants received LZD as part of Uganda’s standardized endTB-based RR-TB regimen, which included bedaquiline, a fluoroquinolone, clofazimine, cycloserine, and LZD; delamanid was used in a minority of cases per national guidelines. CBCs were performed at baseline and scheduled monthly during treatment. Follow-up completion varied due to treatment completion, loss to follow-up, death, and administrative censoring. For analysis, participants were categorized into three groups: the full cohort (*n* = 412) for descriptive epidemiology and composite outcomes; the baseline-CBC cohort (*n* = 245; patients with at least one baseline hematological measurement) for comparing pre-treatment vs. on-treatment values; and the strictly incident cohort (*n* = 81; a subset of the baseline-CBC cohort with documented normal baseline values for hemoglobin, platelet count, and white blood cell count) for estimating the incidence of new events directly attributable to linezolid and for sensitivity analyses of incident adverse events.

Use of antiretroviral therapy (ART) and cotrimoxazole prophylaxis was documented for patients with HIV infection. Information on ART and CD4 cell counts was not consistently available in the records and therefore could not be comprehensively summarized. Cotrimoxazole was excluded from multivariable models due to near-universal exposure among HIV-positive participants. CD4 cell counts were summarized descriptively only.

Data were collected using KoboToolbox, a secure electronic data capture platform that supports offline entry, automated validation rules, skip logic, and standardized coding. Built-in range and consistency checks minimized entry errors. Records were uploaded to a centralized server and reviewed for completeness and internal consistency before analysis [16].

### Study variables

Sociodemographic variables included year of enrollment in care, age, sex, residence, marital status, employment status, and alcohol or cigarette use. Clinical variables included prior tuberculosis treatment, HIV status, and comorbidities such as diabetes mellitus, hypertension, malignancy, hearing impairment, cardiac disease, renal dysfunction, and hepatic dysfunction. Hepatic dysfunction was defined as alanine aminotransferase or aspartate aminotransferase levels greater than two times the laboratory upper limit of normal. Renal dysfunction was defined as serum creatinine exceeding 1.5 times the laboratory upper limit of normal. Laboratory variables included hemoglobin (Hb), platelet counts, and white blood cell (WBC) counts. Treatment-related variables included TB resistance profiles, treatment regimen composition, duration of treatment, time to sputum culture conversion, LZD dose changes, treatment discontinuation, and treatment outcomes. Among HIV-positive participants, antiretroviral therapy status and recent CD4 cell counts were summarized.

### Definition of hematological AEs

Hematological AEs were defined using complete blood count measurements obtained at baseline and during follow-up. Anemia was defined as Hb less than 12 g/dL in women or less than 13 g/dL in men. Thrombocytopenia was defined as a platelet count less than 150 × 10³/µL. Leukopenia was defined as a WBC count less than 3.7 × 10⁹/L. Severity of hematological AEs was graded using the Division of AIDS Table for Grading the Severity of Adult and Pediatric AEs, version 2.1 (July 2017) [17]. Grades 1–2 were categorized as mild to moderate, while Grades 3–4 were categorized as severe.

For analytic purposes, hematological AEs were classified into three categories:

1. **Pre-existing** - an AE (anemia, thrombocytopenia, or leukopenia) documented at baseline in any of the three parameters (hemoglobin, platelet count, or white blood cell count).
2. **First detected after LZD initiation** - the first recorded AE occurring after starting LZD, regardless of whether baseline CBC was available. This category includes patients with missing baseline data and therefore does not represent strictly incident events.
3. **Strictly incident** - a new-onset AE (in any of the three parameters) occurring after LZD initiation only among patients who had documented normal baseline values for all three parameters (i.e., no baseline anemia, thrombocytopenia, or leukopenia). A patient who had, for example, baseline anemia but normal platelet count and then developed thrombocytopenia during follow-up would contribute a strictly incident thrombocytopenia AE (for the platelet lineage) but would **not** contribute a strictly incident composite hematological AE because they had a pre-existing AE at baseline. For the composite outcome of any strictly incident hematological AE, the patient must have been free of all three AEs at baseline and then developed at least one AE during follow-up.

For the primary multivariable analysis, the outcome was defined as the first documented occurrence of any hematological AE (anemia, thrombocytopenia, or leukopenia) after LZD initiation, irrespective of baseline CBC availability.

### Treatment outcomes

Treatment outcomes were classified according to World Health Organization (WHO) definitions for drug-resistant tuberculosis. Cure, treatment failure, relapse, loss to follow-up, and death were defined using standard WHO criteria. Relapse was defined as recurrent tuberculosis after treatment completion; reinfection could not be distinguished due to the absence of genotyping data, and all recurrent cases were classified as relapse. AEs monitored during treatment included hematological, neuro-ophthalmic, hepatic, and other clinically significant toxicities. The composite hematological AEs outcome included both baseline hematological AEs and events detected during follow-up unless otherwise specified.

### Data analysis

All eligible RR-TB patients across the four study sites were included in the analysis. Data were exported from KoboToolbox to Excel and analyzed using STATA version 18. Continuous variables were summarized using means and standard deviations or medians and interquartile ranges, depending on distribution. Descriptive statistics were presented using tables and figures. Hematological AEs were analyzed as both individual hematological AEs and a composite outcome representing the occurrence of any hematological AEs during follow-up. Associations between participant characteristics and hematological AEs were evaluated using bivariable and multivariable models. Severity grading was applied at the hematological AEs level, allowing individuals to contribute multiple graded events.

Associations between participant characteristics and hematological AEs were examined using bivariable analyses. The χ² test was used for categorical variables, while the t test or Mann-Whitney U test was applied for continuous variables, depending on data distribution. Variables with *p* < 0.20 at the bivariable stage were entered into a multivariable modified Poisson regression model with robust standard errors to estimate adjusted prevalence ratios. This approach was selected to provide interpretable effect estimates for common outcomes. Statistical significance was defined as *p* < 0.05. Cases with substantial missing data on key variables were excluded from analyses requiring those variables, and no imputation was performed because the high proportion of missingness (40.5% for baseline CBC) and the non-random pattern of missing data (see Supplementary Table S8) made imputation assumptions (e.g., missing at random) untenable. To assess potential selection bias due to missing baseline CBC data, we compared baseline demographic and clinical characteristics between patients with and without baseline CBC data using χ² tests for categorical variables and t tests for continuous variables; the results are presented in Supplementary Table S8 and described in the Results section. The *p* < 0.20 threshold was used as a screening tool to avoid omitting potentially important confounders, consistent with established practices in exploratory observational studies. To assess the robustness of our findings, we also conducted a sensitivity analysis using a full model that included all a priori selected covariates without p-value screening (Supplementary Table S7). To assess the potential impact of surveillance bias (differential CBC monitoring frequency), we repeated the multivariable Poisson regression including the total number of CBC measurements after LZD initiation as an additional covariate, allowing us to determine whether the association between rural residence and hematological AEs was independent of monitoring intensity. The results of this sensitivity analysis are presented in Supplementary Table S9.

### Ethical considerations

The study was approved by the Mbale Regional Referral Hospital Research and Ethics Committee (Reference Number MRRH-2024-497), with additional administrative clearance from participating hospitals. Informed consent was waived because the study involved retrospective review of routinely collected clinical records. All datasets were de-identified prior to analysis to protect patient confidentiality.

## Results

### Social demographic and clinical characteristics of participants

**Fig. 1:**
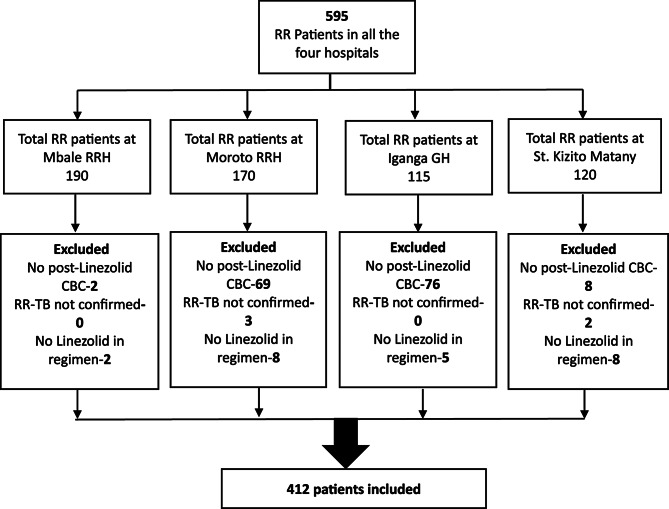
Study flow diagram

The patient selection process is shown in Fig. 1.

The study included 412 participants with a mean age of 40.2 (± 17.5) years. Most participants resided in rural areas (83.5%), and 60.2% were male. Elevated liver enzymes were observed in 22.7%. Among participants with available substance use data (*n* = 313), 46.3% reported cigarette smoking and 49.8% reported alcohol use. Sociodemographic and clinical characteristics are summarized in Table 1. Baseline hematological measurements were available for 243 patients for Hb, 226 for platelet count, and 230 for WBC count. Comparison of patients with and without baseline CBC data.

**Table 1:** Baseline characteristics of the study population of RR-TB patients on LZD-based regimens (*n* = 412)

Characteristic	Frequency	Percentage
Age (Mean and Standard Deviation)	40.2 (± 17.5)	
Residence		
Rural	344	83.5
Urban	68	16.5
Sex		
Female	164	39.8
Male	248	60.2
Pregnancy and breastfeeding status among female participants (*n* = 164)		
Breastfeeding	12	7.3
Not pregnant	146	89.0
Pregnant	6	3.7
Nature of Employment		
Employed	40	9.7
Self-employed	71	17.2
Unemployed	301	73.1
Marital Status		
Divorced	38	9.4
Married	270	66.8
Single	71	17.6
Widowed	25	6.2
Cigarette smoking (*n* = 313)		
No	168	53.7
Yes	145	46.3
Alcohol use (*n* = 313)		
No	157	50.2
Yes	156	49.8
Renal dysfunction (*n* = 271)		
Known renal dysfunction	8	3.0
Unknown renal dysfunction	263	97.0
Hepatic dysfunction (*n* = 269)		
Elevated liver enzymes	61	22.7
Unknown/No elevated liver enzymes	208	77.3
HIV status		
Negative	351	85.2
Positive	61	14.8
Among HIV-positive (*n* = 61)		
On ART	58	95.1
Not on ART	3	4.9
Recent CD4 available	30	49.2
Median CD4 (IQR), cells/µL	346 (200–505)	
Any documented AEs during follow-up (*n* = 271)		
No	74	27.3
Yes	197	72.7
Any change in treatment plan during follow-up (*n* = 272)		
No	232	85.3
Yes	40	14.7
Baseline complete blood count available	245	59.5
Baseline Hb (*n* = 243)		
Median (IQR), g/dL	12.4 (10.8–13.5)	
Baseline platelet count (*n* = 226)		
Median (IQR), ×10³/µL	219 (166–329)	
Baseline WBC count (*n* = 230)		
Median (IQR), ×10⁹/L	5.3 (4.3–7.1)	

Note: Percentages are calculated using the number of participants with available data for each variable. Pregnancy and breastfeeding status were assessed among female participants only. Not all baseline comorbidities and follow-up documentation variables were available for the full cohort. Baseline CBC was defined as the availability of at least one hematological parameter, resulting in 245 participants with baseline hematologic data. Hb was available for 243 participants, platelet count for 226 participants, and WBC count for 230 participants. Differences in denominators reflect missing values for specific CBC components rather than the absence of baseline CBC. Cotrimoxazole prophylaxis was documented in 95.1% (58/61) of HIV-positive patients. Pregnancy and breastfeeding status were assessed among female participants (*n* = 164)

As shown in Supplementary Table S8, patients without baseline CBC data (*n* = 167) differed significantly from those with baseline CBC data (*n* = 245) in several characteristics. They were more likely to reside in rural areas (88.6% vs. 80.0%, *p* = 0.021), less likely to report cigarette smoking (37.8% vs. 67.1%, *p* < 0.001), and had a different marital status distribution (*p* = 0.007), with a higher proportion of married individuals (70.1% vs. 62.4%) and a lower proportion of divorced or widowed individuals (10.2% vs. 22.0%). No significant differences were observed in age, sex, or HIV status. These differences are reported in Supplementary Table S8. Among the 245 participants with baseline hematologic data available, 98 (40.0%) developed at least one incident hematological AE. LZD was administered at a standard dose of approximately 600 mg once daily in 95.4% (393/412) of patients, with reduced doses of 300 mg in 2.9% (12/412) and 150 mg in 1.7% (7/412). The mean duration of LZD exposure was 5.9 ± 2.0 months, with a median of 6 months (range 1–12 months).

### Occurrence of hematological AEs during treatment

Among 412 patients, 62.9% (259/412) had at least one hematological AE documented at baseline or during follow-up. At baseline, 39.8% (164/412) had at least one hematological abnormality (Supplementary Table S1). During follow-up, 36.4% (150/412) had a first-detected AE after LZD initiation (note that some patients with baseline abnormalities also developed new events; the sum exceeds 62.9% because categories overlap). Events first detected after LZD initiation include participants lacking complete baseline hematological data and therefore do not represent strictly incident hematological AEs.

Among patients with available baseline hematological data, significant declines were observed between baseline and worst on-treatment hematological values. Median Hb decreased from 12.4 g/dL at baseline to 11.7 g/dL during follow-up (Wilcoxon signed-rank test, *p* < 0.001). Median WBC count declined from 5.29 × 10⁹/L to 3.30 × 10⁹/L (*p* < 0.001), while median platelet count decreased from 219 × 10⁹/L to 132 × 10⁹/L (*p* < 0.001).

**Fig. 2:**
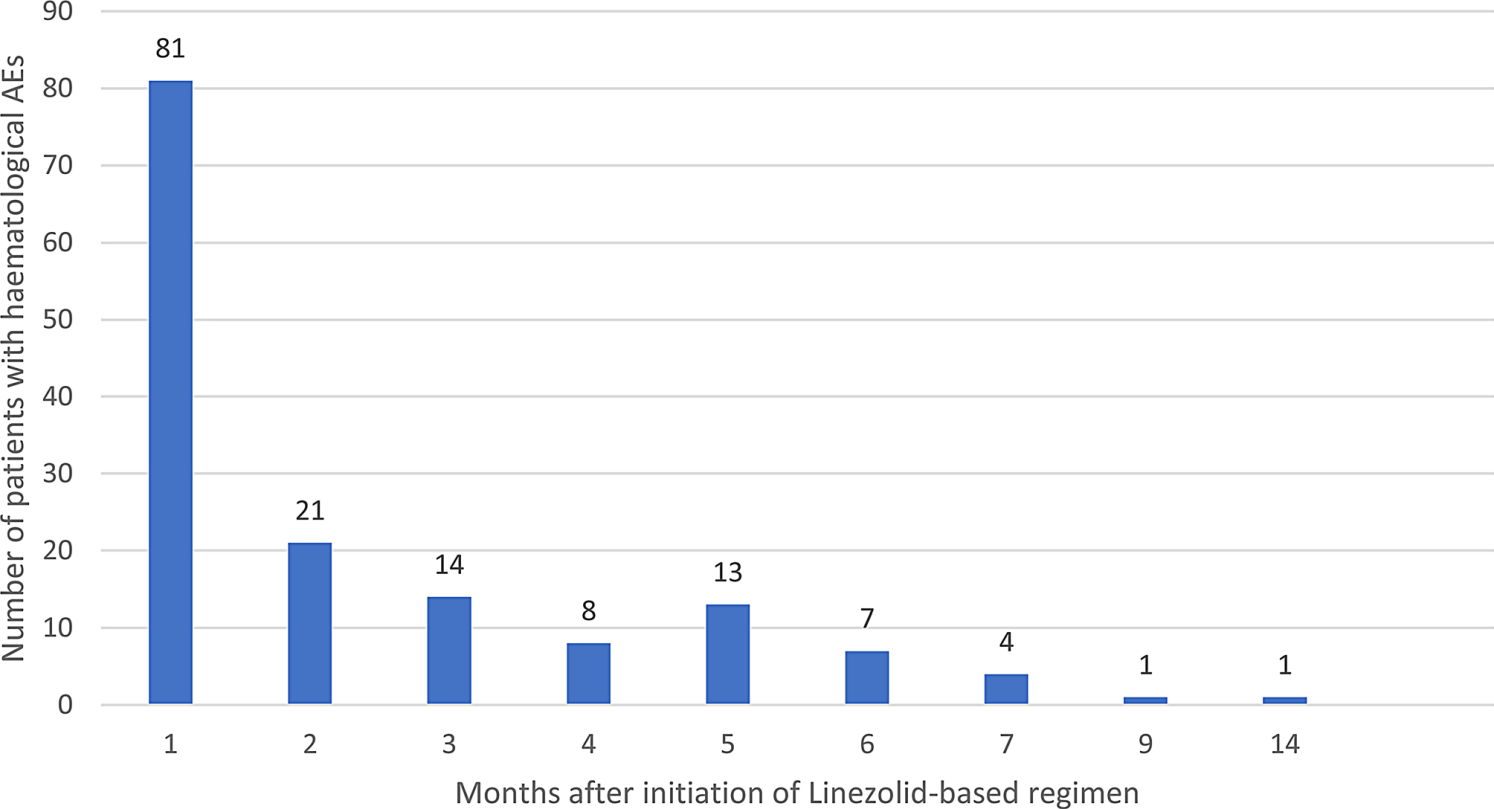
Monthly distribution of hematological AEs first documented among RR-TB patients receiving LZD-based regimens (*n* = 150). Note: Monthly distribution of hematological AEs first documented among RR-TB patients receiving LZD-based regimens (*n* = 150 events). Bars represent absolute numbers of first-detected events. The monthly decline reflects both decreasing number of patients under active follow-up and the programmatic CBC schedule. Corresponding incidence proportions (events per patients with CBC available) were: Month 1: 19.7%; Month 2: 8.0%; Month 3: 5.5%; Month 4: 3.5%; Month 5: 3.1%; Month 6: 2.3% (see Supplementary Table S2 for monthly denominators)

Among patients with hematological AEs first detected after LZD initiation, 54.0% (81/150) were identified in month 1, with additional events detected during later months of follow-up. The monthly distribution of first-detected AEs is shown in Fig. 2. Supplementary Table S2 provides the monthly denominators (number of patients with available CBC results) corresponding to each month in Fig. 2. When all hematological measurements across baseline and follow-up were considered, thrombocytopenia was the most frequently recorded hematological AE, occurring in 56.6% (233/412) of participants, followed by leukopenia in 56.3% (232/412). Anemia was documented in 43.7% (180/412). These frequencies reflect both baseline hematological AEs and newly detected events.

Among the 245 participants with any baseline hematological data, 98 (40.0%) developed at least one new hematological AE after LZD initiation (including events in patients who had a pre-existing AE in a different lineage). Among the 81 patients who had no baseline hematological AEs in any of the three parameters (the strictly incident cohort), 34 (42.0%) developed at least one strictly incident hematological AE during follow-up. This proportion did not differ significantly from the 39.0% (64/164) incidence of new AEs among those with at least one pre-existing AE at baseline (*p* = 0.657). Note that in the latter group, a new AE could occur in a lineage that was normal at baseline (e.g., baseline anemia followed by new thrombocytopenia). In lineage-specific analyses across the full cohort, first-detected anemia occurred in 21.4% (88/412), thrombocytopenia in 14.8% (61/412), and leukopenia in 13.8% (57/412) of patients. Longitudinal follow-up showed worsening hematological AEs in a subset of patients. Progression to a more severe anemia grade occurred in 27.2% (112/412) of the full cohort, worsening thrombocytopenia in 14.8% (61/412), and worsening leukopenia in 13.8% (57/412). Overall, 32.8% (135/412) experienced worsening of at least one hematological parameter.

Because detection of hematological AEs depends on how frequently CBCs are performed, we first compared monitoring intensity by residence. Rural patients had significantly fewer total CBCs after linezolid initiation (median 4, IQR 0–6) compared to urban patients (median 5, IQR 3–6; *p* = 0.009, Mann-Whitney U test). Rural patients were also more likely to have low monitoring (< 3 CBCs) (45.3% vs. 22.1%, *p* < 0.001). Monthly CBC completion rates were similar for months 1–5 but differed at month 6 (80.5% for rural vs. 65.9% for urban, *p* = 0.039). However, the testing rate (CBCs per month of follow-up) did not differ significantly (mean 0.79 vs. 0.76, *p* = 0.133).

We then repeated the multivariable analysis adjusting for the total number of CBCs after LZD initiation. In this model (Supplementary Table S9), rural residence remained significantly associated with a higher incidence of hematological AEs (IRR 1.10, 95% CI 1.00-1.20, *p* = 0.041). A higher number of CBCs was strongly associated with increased AE detection (IRR 1.08 per additional CBC, 95% CI 1.05–1.12, *p* < 0.001).

Bivariate analyses, Table 2, assess factors associated with hematological AEs (thrombocytopenia, leukopenia, and anemia) among patients receiving LZD-containing RR-TB regimens in Uganda.

**Table 2:** Comparisons of sociodemographic and clinical characteristics between patients on LZD-containing regimens with and without hematological AEs. (*n* = 412)

Variable	Thrombocytopenia		*P*-Value	Leukopenia		*P*-Value	Anemia		*P*-Value	
	No (*n* = 179)	Yes (*n* = 233)		No (*n* = 180)	Yes (*n* = 232)		No (*n* = 232)	Yes (*n* = 180)		
Age, Mean (± SD)	39.2 (± 17.5)	41.1(± 17.4)	0.275	39.0 (± 17.6)	41.2 (± 17.3)	0.204	38.3(± 17.0)	42.7 (± 17.7)	0.011	
Treatment duration, mean (± SD)	5.9 (± 1.5)	5.9 (± 2.4)	0.884	5.9 (± 1.5)	5.9 (± 2.4)	0.884	5.9 (± 1.9)	6.1 (± 2.2)	0.591	
Residence			< 0.001			< 0.001			0.006	
Rural	167 (93.3)	177 (76.0)		168 (93.3)	176 (75.9)		204 (87.9)	140 (77.8)		
Urban	12 (6.7)	56 (24.0)		12 (6.7)	56 (24.1)		28 (12.1)	40 (22.2)		
Sex			0.243			0.198			0.018	
Female	77 (43.0)	87 (37.3)		78 (43.3)	86 (37.1)		104 (44.8)	60 (33.3)		
Male	102 (57.0)	146 (62.7)		102 (56.7)	146 (62.9)		128 (55.2)	120 (66.7)		
HIV status			0.124			0.114			0.020	
Negative	158 (88.3)	193 (82.8)		159 (88.3)	192 (82.8)		206 (88.8)	145 (80.6)		
Positive	21 (11.7)	40 (17.2)		21 (11.7)	40 (17.2)		26 (11.2)	35 (19.4)		
Cigarette smoking			< 0.001			< 0.001			0.004	
Yes	56 (35.4)	89 (57.4)		56 (35.4)	89 (57.4)		101 (52.9)	44 (36.1)		
No	66 (42.6)	102 (64.6)		66 (42.6)	102 (64.6)		90 (47.1)	78 (63.9)		
Alcohol use			0.283			0.283			0.115	
No	73 (47.1)	84 (53.2)		82 (52.9)	84 (53.2)		89 (46.6)	68 (55.7)		
Yes	82 (52.9)	74 (46.8)		73 (47.1)	74 (46.8)		102 (53.4)	54 (44.3)		
Renal Dysfunction			0.246			0.239			0.573	
Known Renal Dysfunction	0 (0.0)	8 (3.4)		0 (0.0)	8 (3.4)		2 (2.2)	6 (3.4)		
Unknown renal dysfunction	38 (100.0)	225 (96.6)		39 (100.0)	224 (96.6)		91 (97.8)	172 (96.6)		
Hepatic Dysfunction			0.005			0.004			0.099	
Elevated liver enzymes	2 (5.1)	59 (25.7)		2 (5.0)	59 (25.8)		26 (28.6)	35 (19.7)		
Unknown/No elevated liver enzymes	37 (94.9)	171 (74.3)		38 (95.0)	170 (74.2)		65 (71.4)	143 (80.3)		
Documentation of AEs of any type during follow-up			0.976			0.941			0.002	
No	11 (27.5)	63 (27.3)		11 (26.8)	63 (27.4)		36 (39.1)	38 (21.2)		
Yes	29 (72.5)	168 (72.7)		30 (73.2)	167 (72.6)		56 (60.9)	141 (78.8)		
Changes in treatment plan during follow-up			0.391			0.359			0.055	
No	36 (90.0)	196 (84.8)		37 (90.2)	195 (84.8)		84 (91.3)	148 (82.7)		
Yes	4 (10.0)	35 (15.2)		4 (9.8)	35 (15.2)		8 (8.7)	31 (17.3)		

Note: This comparison includes all documented hematological AEs, including those present at baseline and those newly detected during follow-up. Percentages reflect column proportions (i.e., within each hematological AE category, the distribution of each characteristic). Denominators for categorical variables vary due to missing data: cigarette smoking and alcohol use (*n* = 313), renal dysfunction (*n* = 271), hepatic dysfunction (*n* = 269), documentation of AEs (*n* = 271), and changes in treatment plan (*n* = 272). To calculate the prevalence of each AE within an exposure group (e.g., percentage of urban residents with thrombocytopenia), divide the cell count by the row total (e.g., 56/68 = 82.4%)

Thrombocytopenia occurred in a higher proportion of urban residents (82.4%, 56/68) than rural residents (51.5%, 177/344) (*p* < 0.001). It was also more common among smokers (61.4%, 89/145) than non-smokers (33.3%, 56/168) (*p* < 0.001). Among participants with hepatic dysfunction, thrombocytopenia occurred in 96.7% (59/61) vs. 73.6% (171/232) in those without (*p* = 0.005). Leukopenia showed similar patterns: urban residents (82.4%, 56/68) vs. rural (51.2%, 176/344) (*p* < 0.001); smokers (61.4%, 89/145) vs. non-smokers (33.3%, 56/168) (*p* < 0.001); and hepatic dysfunction (96.7%, 59/61) vs. without (73.3%, 170/232) (*p* = 0.004). HIV-positive participants had leukopenia in 65.6% (40/61) vs. HIV-negative 54.7% (192/351) (*p* = 0.114, not significant). Anemia was more common among urban residents (58.8%, 40/68) than rural (40.7%, 140/344) (*p* = 0.006). Among smokers, anemia occurred in 30.3% (44/145) vs. 46.4% (78/168) in non-smokers (*p* = 0.004). Anemia was also more frequent in males (48.4%, 120/248) than females (36.6%, 60/164) (*p* = 0.018), and in PLHIV (57.4%, 35/61) vs. HIV-negative (41.3%, 145/351) (*p* = 0.020). Participants with anemia had a higher mean age than those without (42.7 vs. 38.3 years, *p* = 0.011). Anemia was also more common among patients with documented AEs during follow-up (78.8%, 141/179) vs. those without (21.2%, 38/179) (*p* = 0.002).

Combined hematological AE analyses were restricted to post-baseline measurements. Pancytopenia, defined as concurrent anemia, thrombocytopenia, and leukopenia at any time during follow-up, occurred in 5.6% (23/412) of patients. Bicytopenia, defined as the concurrent presence of any two hematological AEs during follow-up, occurred in 12.6% (52/412) of patients.

Severity of hematological AEs was graded according to DAIDS criteria, as summarized in Table 3. Across all hematological AE occurrences, 70.3% (182/259) of recorded events were DAIDS graded (Grades 1–4), with most classified as Grade 1 or Grade 2. LZD dose modification or interruption occurred in 13.7% (25/182) of graded event occurrences. Among Grade 3–4 event occurrences, 14.6% (18/123) required dose modification or interruption. Most patients with Grades 3–4 hematological AEs continued LZD without permanent discontinuation. Treatment interruptions during follow-up were more frequently attributed to non-hematological AEs, particularly neuro-ophthalmic AEs. **At the patient level**, among the 259 patients with any hematological AE, 123 (47.5%) experienced at least one Grade 3–4 event. Of these 123 patients, 18 (14.6%) required LZD dose modification or interruption (consistent with the event-level proportion). Permanent LZD discontinuation due to hematological AEs alone occurred in 3 patients (2.4% of those with Grade 3–4 events), while mixed hematological and neurological AEs led to discontinuation in an additional 4 patients (3.3%). The majority of patients with severe hematological AEs (105/123, 85.4%) continued LZD without dose modification.

**Table 3:** Severity grading of hematological AEs occurrences using DAIDS criteria

Adverse Event	Grade 1 *n* (%)	Grade 2 *n* (%)	Grade 3 *n* (%)	Grade 4 *n* (%)	Total (Grades 1–4)
Thrombocytopenia	94 (66.2)	15 (10.6)	19 (13.4)	14 (9.9)	142
Leukopenia	54 (38.6)	68 (48.6)	18 (12.9)	0	140
Anemia	114 (63.3)	37 (20.6)	15 (8.3)	14 (7.8)	180

Note: Counts represent hematological AEs occurrences rather than unique patients, as some individuals experienced more than one hematological AEs or multiple graded events over time

Patients residing in rural areas were 1.6 times as likely to develop the composite hematological AE outcome compared to urban residents (aPR 1.60, 95% CI 1.11–2.33, *p* = 0.012). Divorced or widowed patients were 4.1 times as likely to develop the composite hematological AE outcome compared to those who never married (aPR 4.10, 95% CI 1.58–10.77, *p* = 0.004). Patients who smoked had a higher likelihood of developing the composite hematological AE outcome (aPR 1.70, 95% CI 1.28–2.80, *p* = 0.005). However, in a sensitivity analysis adjusting for monitoring intensity (Supplementary Table S9), cigarette smoking was not associated with hematological AEs (IRR 0.98, 95% CI 0.88–1.09, *p* = 0.678). As shown in Table 4.

**Table 4:** Factors associated with the occurrence of at least one hematological AE among RR-TB patients receiving LZD-based regimens

Variable	cPR (95% CI)	*P*-Value	aPR (95% CI)	*P*-Value
**Residence**				
Urban	1		1	
Rural	2.60 (1.52–4.45)	0.001	1.60 (1.11–2.33)	**0.012**
**Nature of Employment**				
Employed	1		1	
Self-employed	1.40 (0.63–2.98)	0.432	1.50 (0.56–3.95)	0.425
Unemployed	0.70 (0.34–1.27)	0.214	0.50 (0.23–1.23)	0.139
**Marital Status**				
Single/never married	1		1	
Divorced/widowed	3.00 (1.49–6.09)	0.002	4.10 (1.58–10.77)	**0.004**
Married	1.20 (0.70–2.07)	0.492	1.10 (0.50–2.35)	0.848
**Cigarette smoking**				
Yes	2.70 (1.24–4.71)	0.008	1.70 (1.28–2.80)	**0.005**
No	1		1	
**HIV status**				
Negative	1		1	
Positive	1.50 (0.88–2.61)	0.136	1.20 (0.57–2.39)	0.683
**Alcohol use**				
No	1		1	
Yes	1.10 (0.68–1.68)	0.782	1.10 (0.64–1.84)	0.766
**Previous history of TB treatment**				
No	1		1	
Yes	1.00 (0.66–1.53)	0.971	1.30 (0.74–2.15)	0.404

To assess the robustness of our findings, we performed three sensitivity analyses. First, repeating the multivariable analysis in the strictly incident cohort (*n* = 81) yielded associations directionally consistent with the primary analysis, though the small sample size limited precision (Supplementary Table S5). Second, restricting the analysis to HIV-negative patients (*n* = 269) to address potential confounding by cotrimoxazole, the association between rural residence and incident hematological adverse events remained robust (aPR 1.10, 95% CI 1.00-1.21, *p* = 0.045) (Supplementary Table S6). Third, we used a full model that included all a priori selected covariates without p-value screening; results were consistent with the primary analysis, with rural residence (aPR 1.34, 95% CI 1.09–1.64, *p* = 0.005), cigarette smoking (aPR 1.67, 95% CI 1.35–2.07, *p* < 0.001), and married status (aPR 0.66, 95% CI 0.53–0.84, *p* = 0.001) independently associated with hematological adverse events (Supplementary Table S7). In this full model, married status showed a protective association, whereas divorced/widowed status was not significant. Sensitivity analyses restricted to HIV-negative patients (Supplementary Table S6) and using a full model without p-value screening (Supplementary Table S7) confirmed that rural residence remained associated with hematological AEs (aPR 1.10, 95% CI 1.00-1.21 and aPR 1.34, 95% CI 1.09–1.64, respectively).

### Hematological AEs and treatment outcome among patients with RR-TB who received LZD-based regimen

Treatment regimen modification occurred in 19.9% (54/272) of patients receiving LZD. Unfavorable treatment outcomes were not more frequent among patients who required dose modification compared with those who did not (9.3% (5/54) vs. 16.1% (35/218)). All permanent LZD discontinuations occurred among patients who underwent dose modification, consistent with regimen adjustment driven by AEs, not by treatment failure. Loss to follow-up was more common among patients who did not require dose modification (Table 5).

**Table 5:** Treatment modifications and outcomes

Variable	Unfavorable *n* (%)	Favorable *n* (%)	Total *n*	*P*-value
**Regimen modification**				
Yes	5 (9.3%)	49 (90.7%)	54	0.27
No	35 (16.1%)	183 (83.9%)	218	
**Treatment discontinuation**				
Yes	2 (33.3%)	4 (66.7%)	6	0.66
No	35 (16.1%)	183 (83.9%)	218	

Note: Same denominators for both “No” groups because all discontinuations were in the modified group

Among patients with hematological AEs, treatment interruption occurred in 15.1% (39/259) and was observed across anemia, thrombocytopenia, and leukopenia, with similar proportions across hematological AEs categories (Supplementary Table S3). LZD management strategies varied by hematological AEs type and severity. Permanent discontinuation occurred in 9.8% (5/51) of patients with anemia and 8.9% (16/180) of patients with leukopenia (see management details in Supplementary Tables S3 and S4). Temporary interruption or dose reduction was uncommon, occurring in 2.0% (1/51) of anemia cases and 3.3% (6/180) of leukopenia cases. Most patients with hematological AEs continued LZD without regimen modification. Management actions are summarized in Supplementary Table S4.

LZD was discontinued in 7.8% (32/412) of patients. Neuro-ophthalmic AEs accounted for 68.8% (22/32) of discontinuations. Hematological AEs alone accounted for 9.4% (3/32), while mixed hematological and neurological AEs accounted for 12.5% (4/32). Overall, hematological AEs were documented in 21.9% (7/32) of all LZD discontinuations. All proportions refer to the subset of patients who discontinued LZD.

Among patients who developed hematological AEs during treatment (*n* = 259), 83.0% (215/259) achieved cure or completion, 1.9% (5/259) died, and 15.1% (39/259) were lost to follow-up. In contrast, among patients without hematological AEs (*n* = 153), 93.5% (143/153) achieved cure or completion, 0.7% (1/153) died, and 5.9% (9/153) were lost to follow-up.

Patients with any hematological AEs had a higher cumulative risk of unfavorable treatment outcome, 17.0% (44/259) versus 6.5% (10/153), RR 2.60 (95% CI 1.35 to 5.01, *p* = 0.002). Pancytopenia was strongly associated with unfavorable outcomes, occurring in 43.5% (10/23) of patients with pancytopenia compared with 11.3% (44/389) of those without (RR 3.12, 95% CI 1.72 to 5.65, *p* < 0.001). Bicytopenia during follow-up was also associated with increased risk of unfavorable outcomes, observed in 26.3% (21/52) of affected patients compared with 9.9% (33/360) among those without bicytopenia first detected after LZD initiation (RR 2.64, 95% CI 1.59 to 4.40, *p* < 0.001). Severe lineage-specific hematological AEs were not associated with unfavorable outcomes. LZD dose modification or interruption was not associated with unfavorable outcome (RR 0.59, 95% CI 0.25 to 1.39, *p* = 0.207).

**Fig. 3:**
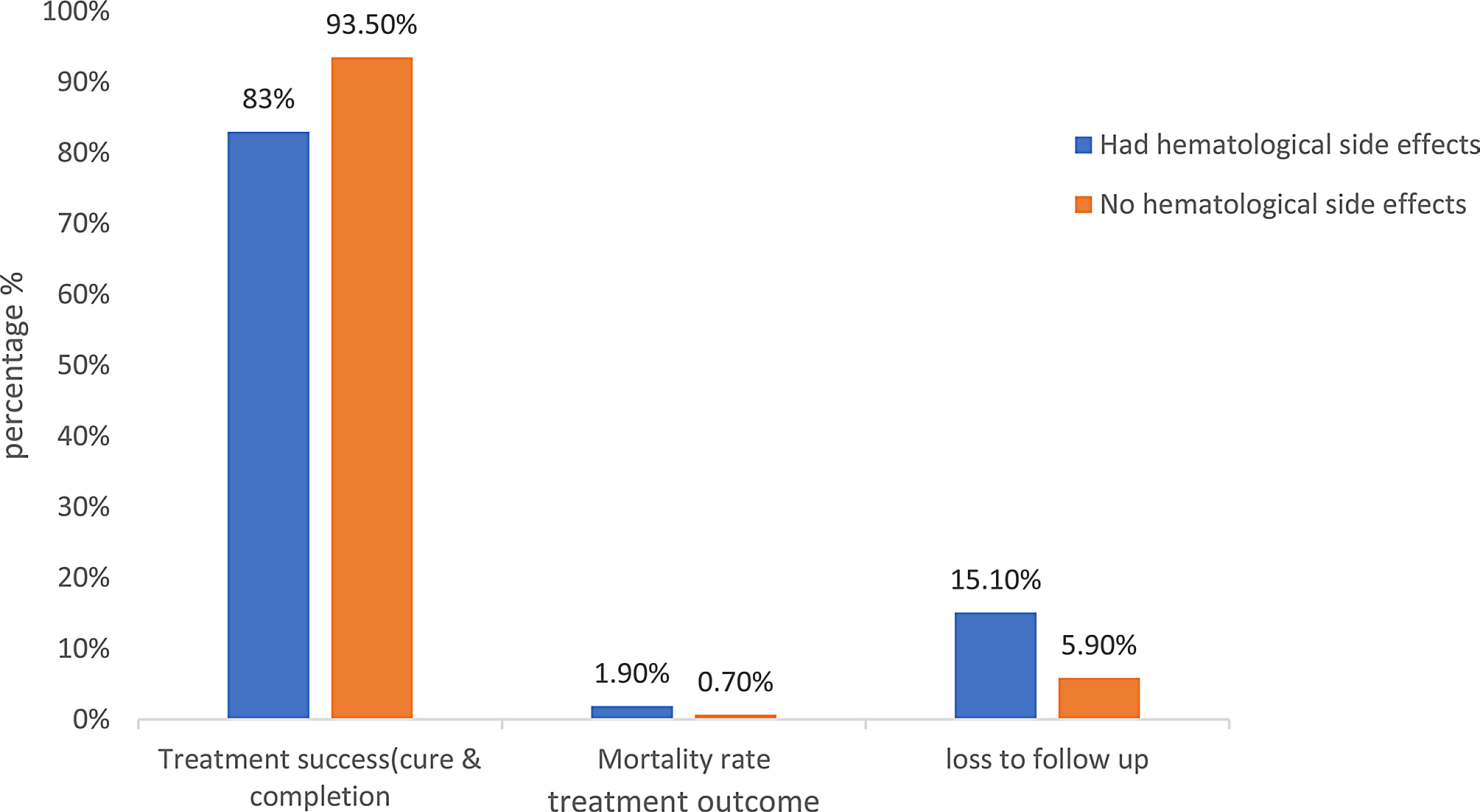
Treatment outcomes by hematological AEs status. Note: Fig. 3 shows treatment outcomes among RR-TB patients receiving LZD-based regimens, stratified by hematological AEs status. Percentages represent row proportions within each group

### Factors associated with treatment success among patients with RR-TB who received LZD-based regimen

HIV-positive patients had a higher likelihood of treatment success compared with HIV-negative patients (aPR 1.10; 95% CI 1.02–1.20). Patients who did not report alcohol use were more likely to achieve treatment success than alcohol users (aPR 1.90; 95% CI 1.02–2.21). Unemployed patients had a lower likelihood of treatment success compared with employed patients (aPR 0.90; 95% CI 0.77–0.96). These findings are summarized in Table 6.

**Table 6:** Factors associated with treatment success among patients with RR-TB who received LZD-based regimen

Variable	cPR (95% CI)	*P*-Value	aPR (95% CI)	*P*-Value
Residence				
Urban				
Rural	1.10 (1.00–1.17)	0.057	1.00 (0.93–1.17)	0.454
Marital Status				
Single/never married				
Divorced/widowed	0.90 (0.81–1.05)	0.198	1.20 (0.85–1.23)	0.832
Married	0.90 (0.87–1.03)	0.206	1.60 (0.86–2.12)	0.748
HIV status				
Negative				
Positive	1.20 (1.10–1.22)	< 0.001	1.10 (1.02–1.20)	**0.012**
Alcohol use				
Yes				
No	1.20 (1.08–1.29)	< 0.001	1.90 (1.02–2.21)	**0.021**
Cigarette smoking				
Yes				
No	1.10 (1.03–1.22)	0.012	1.50 (0.93–2.10)	0.829
Nature of Employment				
Employed				
Self-employed	1.00 (0.94–1.11)	0.585	1.10 (0.97–1.20)	0.148
Unemployed	0.90 (0.81–0.96)	0.003	0.90 (0.77–0.96)	**0.008**
Previous history of TB treatment				
No				
Yes	1.20 (0.9–1.06)	0.612	1.40 (0.953–2.14)	0.368
Having hematological AEs				
No				
Yes	0.80 (0.77–0.93)	< 0.001	0.90 (0.77–1.03)	0.108

## Discussion

This multicenter cohort study demonstrates that hematological AEs are a frequent complication of LZD-based regimens for RR-TB in Uganda. A substantial proportion (54.0%) of incident hematological AEs were detected within the first month of treatment. This early-onset pattern is consistent with previous reports of LZD-associated hematological AEs [13, 18], and aligns with the exposure-threshold model wherein initial drug levels drive toxicity risk [18, 19]. Together, these findings highlight that hematological AEs are common and often occur early during LZD-based RR-TB treatment.

The burden of hematological AEs in this study was higher than rates reported in RR-TB cohorts from South Africa, Indonesia, China, and Nigeria [6, 15, 20, 21]. This discrepancy may be associated with variations in LZD dosing strategies, duration of exposure, intensity of longitudinal CBC monitoring, and underlying patient risk profiles. The high frequency of cigarette smoking, rural residence, and nutritional vulnerability in this cohort are factors that may be linked to the elevated rates observed.

Our analysis identified rural residence and divorced or widowed marital status as independent factors associated with hematological AEs. Rural residence was strongly linked to a higher prevalence of AEs, a pattern documented in other resource-limited settings where distance limits consistent clinical and laboratory monitoring [6, 21]. In decentralized care models such as Uganda’s, rural patients may encounter structural barriers including transportation limitations, nutritional deficits, and inconsistent laboratory access, factors that can increase susceptibility to treatment-related complications [22]. The association between divorced or widowed marital status and hematological AEs likely reflects underlying social and economic vulnerability. Marital disruption can reduce social support and financial stability, potentially affecting treatment engagement and nutritional status, which may contribute to increased susceptibility to complications. Similar relationships between social disadvantage and adverse TB outcomes have been reported in prior cohorts [6, 21–24].

The bivariate analyses in Table 6 include both baseline and follow-up AEs, whereas the multivariable model (Table 1) specifically examines AEs first detected after LZD initiation. This difference in outcome definition explains the apparent reversal.

Cigarette smoking was associated with hematological AEs in the primary analysis (aPR 1.70, 95% CI 1.28–2.80, *p* = 0.005), but this association was not reproduced in a sensitivity analysis adjusting for monitoring intensity (IRR 0.98, 95% CI 0.88–1.09, *p* = 0.678; Supplementary Table S9). The inconsistency suggests that the observed association may be confounded by unmeasured factors such as socioeconomic status, nutritional deficits, or other lifestyle variables that we could not adequately adjust for. Potential biological mechanisms including oxidative stress and bone marrow suppression have been proposed [18, 25], but these pathways were not directly evaluated. In this cohort, smoking was not associated with severe (DAIDS grade 3–4) hematological AEs or with LZD discontinuation. Therefore, the finding regarding smoking should be interpreted with caution and does not necessarily imply a causal relationship.

The temporal pattern of hematological AEs-with a majority detected early and others emerging later-aligns with previous reports describing both early and late-onset hematological AEs associated with LZD. In this study, the presence of a hematological AE at baseline was not predictive of developing a new incident event during treatment.

Hematological AEs were associated with higher risk of unfavorable treatment outcomes and increased loss to follow-up in this cohort, while mortality remained low and comparable between groups. This pattern suggests hematological AEs may be associated with treatment interruption rather than short-term mortality, aligning with other RR-TB studies where LZD-associated AEs drive discontinuation [21]. Notably, severe hematological AEs accounted for few LZD discontinuations; neuro-ophthalmic events were the predominant cause, consistent with literature describing them as less reversible and more likely to require permanent drug withdrawal [18]. The higher rate of loss to follow-up among patients with hematological AEs likely reflects the cumulative burden of treatment distress, increased clinic visits, and treatment interruptions [22]. This mirrors challenges in other RR-TB programs in Sub-Saharan Africa where treatment-related AEs drive care disengagement [6, 18].

The high overall treatment success observed in this cohort is notable given the clinical complexity of RR-TB and the hematological AEs profile of LZD-based regimens. This favorable outcome likely reflects programmatic improvements within Uganda’s RR-TB response, including optimized regimen composition, decentralized service delivery, strengthened adherence support, and more systematic patient follow-up. Comparable improvements in treatment outcomes have been reported following the implementation of shorter, fully oral RR-TB regimens in similar settings [12].

Although anemia was more frequent among PLHIV in this cohort (Table 6), PLHIV had higher treatment success compared with HIV-negative patients (aPR 1.10, 95% CI 1.02–1.20). This counterintuitive association should not be interpreted as a protective biological effect of HIV infection. Rather, it likely reflects stronger engagement in care, more intensive clinical follow-up, and the benefits of integrated TB-HIV service delivery, including cotrimoxazole prophylaxis and enhanced adherence support. However, we lacked direct measures of adherence or visit frequency to confirm this hypothesis. Residual confounding by unmeasured factors (e.g., socioeconomic status, nutritional support) cannot be excluded. Similar paradoxical associations have been reported in other TB-HIV cohorts [18, 26].

These findings have important implications for RR-TB care in high-burden settings. The elevated risk of hematological AEs among rural residents, individuals experiencing social vulnerability, and those who smoke suggests that structural barriers, behavioral factors, and access to laboratory monitoring influence AE risk and detection. Collectively, these results support strengthening patient-centered follow-up, expanding access to routine CBC monitoring-particularly during the critical first month-and integrating social support interventions into RR-TB programs. Enhanced surveillance for hematological AEs among socially and geographically vulnerable patients is warranted.

A major strength of this study is its multicenter cohort design across key referral hospitals, enhancing the relevance and generalizability of findings within eastern and northeastern Uganda. The large RR-TB cohort treated with LZD-based regimens improves applicability to routine programmatic practice. Longitudinal follow-up enabled evaluation of early versus late hematological AEs, while repeated CBC measurements allowed detailed characterization of AE timing, severity, and progression, as well as clear differentiation between pre-existing hematological AEs and newly detected (incident) events.

This retrospective cohort study has several limitations. Incomplete clinical documentation included missing baseline CBC data in 40.5% of patients and missing CD4 counts in 50.8% of HIV-positive individuals, limiting our ability to fully characterize pre-existing hematological AEs and immunologic status. As shown in Supplementary Table S8, patients without baseline CBC data differed significantly from those with baseline CBC data in rural residence, smoking, and marital status, suggesting selection bias. Consequently, findings from analyses restricted to the baseline CBC cohort (e.g., the 42.0% strictly incident AE incidence) may not be generalizable to all RR-TB patients. However, the primary composite outcome (any hematological AE during follow-up) included all 412 patients and is less susceptible to this bias. CD4 counts were not incorporated into multivariable models due to the high proportion of missing values. Cotrimoxazole was excluded from multivariable models because its use was near-universal (95.1%) among PLHIV, though residual confounding from unmeasured myelotoxic therapies cannot be excluded. Surveillance bias is a potential concern because rural patients underwent fewer CBC measurements than urban patients (median 4 vs. 5, *p* = 0.009). However, when we adjusted for the total number of CBCs in the multivariable model, rural residence remained independently associated with a higher incidence of hematological AEs (IRR 1.10, *p* = 0.041). This suggests that differential monitoring alone does not explain the higher AE risk in rural patients. Nonetheless, we cannot rule out residual confounding from unmeasured factors such as nutritional status or symptom-driven testing. Relapse and reinfection could not be distinguished due to the absence of genotyping. Our variable selection using a *p* < 0.20 threshold may increase overfitting risk, though a sensitivity analysis using a full model without screening yielded consistent results (Supplementary Table S7). We were unable to account for time-varying LZD exposure or perform time-to-event analyses (e.g., Cox regression) due to the retrospective design, variable monitoring schedules, and the fact that most haematological AEs occurred early, limiting the utility of survival methods. A prospective study with standardised monthly CBCs and predefined AE assessment time points would better address temporal relationships. Important potential confounders such as nutritional status and cumulative LZD exposure were not consistently recorded. The strictly incident cohort (*n* = 81) was small, limiting statistical power for subgroup analyses. As with all retrospective studies, causal inference is limited. Regarding cigarette smoking, residual confounding is likely. Smoking is often correlated with lower socioeconomic status, poor nutrition, and alcohol use, factors we could not fully measure or adjust for. The inconsistency between the primary and sensitivity analyses further indicates that the association may not be robust; thus, smoking should not be interpreted as an independent causal risk factor based on this study alone. Regarding the higher treatment success observed among HIV-positive patients, we lacked data on adherence to clinic visits, ART refill consistency, or intensity of psychosocial support. Therefore, we cannot determine whether the association is causal or reflects unmeasured differences in care engagement.

## Conclusion

Hematological AEs, particularly thrombocytopenia and leukopenia, are common in Ugandan RR-TB patients receiving linezolid, with many occurring early. They were associated with rural residence and divorced or widowed status. These events correlated with lower treatment success and higher loss to follow-up but not with increased mortality. The findings support enhanced CBC monitoring and integrated strategies to address social and behavioral risks in RR-TB care.

## Supplementary Information

Below is the link to the electronic supplementary material.

Supplementary Material 1

Supplementary Material 2

Supplementary Material 3

Supplementary Material 4

Supplementary Material 5

Supplementary Material 6

Supplementary Material 7

Supplementary Material 8

Supplementary Material 9

## Data Availability

The datasets used during this study are available from the corresponding author upon reasonable request.

## Abbreviations

AEs: Adverse Events
ALT: Alanine Aminotransferase
AST: Aspartate Aminotransferase
aPR: Adjusted Prevalence Ratio
CBC: Complete Blood Count
CI: Confidence Interval
DAIDS: Division of AIDS
Hb: Hemoglobin
LZD: Linezolid
MDR-TB: Multidrug-Resistant Tuberculosis
RR-TB: Rifampicin-Resistant Tuberculosis
WBC: White Blood Cells
WHO: World Health Organization
