# Supplementary material for: Prevalence and factors associated with hematological adverse events in RR-TB patients on linezolid-based regimens in Uganda: a multicenter retrospective cohort study
Source: BMC Infect Dis. 2026 Apr 30;26:1176. doi: 10.1186/s12879-026-13405-4 (PMC13289349; doi:10.1186/s12879-026-13405-4)
Supplement: Supplementary file 1 — Supplementary Material 1 [file 12879_2026_13405_MOESM1_ESM.pdf]

Supplementary Table S1. Baseline hematological abnormalities (pre-existing adverse events) among RR-TB patients (n=412)

Hematological abnormality	Number with available baseline data	Number with abnormality	Prevalence (among those with data)
Anemia (Hb <12/13 g/dL)	243	134	55.1%
Thrombocytopenia (platelets $<150\times10^3/\mu L$)	226	44	19.5%
Leukopenia (WBC $<3.7\times10^9/L$)	230	31	13.5%
**Any abnormality (composite)**	**412 (full cohort)**	**164**	**39.8%**

Note: Denominators vary because baseline complete blood count components were not available for all patients. The composite "any abnormality" uses available data for each component (i.e., a patient was counted if any available baseline measurement met the criterion).
