# Supplementary material for: Prevalence and factors associated with hematological adverse events in RR-TB patients on linezolid-based regimens in Uganda: a multicenter retrospective cohort study
Source: BMC Infect Dis. 2026 Apr 30;26:1176. doi: 10.1186/s12879-026-13405-4 (PMC13289349; doi:10.1186/s12879-026-13405-4)
Supplement: Supplementary file 2 — Supplementary Material 2 [file 12879_2026_13405_MOESM2_ESM.pdf]

Supplementary Table S2: Monthly CBC Monitoring Denominators

Follow up month	Number with CBC
**Baseline**	245
**Month 1**	172
**Month 2**	170
**Month 3**	162
**Month 4**	143
**Month 5**	141
**Month 6**	134
**Month 7**	106
**Month 8**	89
**Month 9**	50
**Month 10**	32
**Month 11**	26
**Month 12**	14
**Month 13**	11
**Month 14**	9
**Month 15**	6
**Month 16**	5
**Month 17**	3
**Month 18**	1
