# Supplementary material for: Prevalence and factors associated with hematological adverse events in RR-TB patients on linezolid-based regimens in Uganda: a multicenter retrospective cohort study
Source: BMC Infect Dis. 2026 Apr 30;26:1176. doi: 10.1186/s12879-026-13405-4 (PMC13289349; doi:10.1186/s12879-026-13405-4)
Supplement: Supplementary file 3 — Supplementary Material 3 [file 12879_2026_13405_MOESM3_ESM.pdf]

Supplementary Table S3. Treatment Interruption by Hematological Adverse Event Type

Adverse event type	No interruption n (%)	Treatment interruption n (%)	Total
**Anemia**	45 (88.2)	6 (11.8)	51
**Thrombocytopenia**	211 (85.1)	37 (14.9)	248
**Leukopenia**	158 (87.8)	22 (12.2)	180
**Any hematological adverse event**	220 (84.9)	39 (15.1)	259

Note: Treatment interruption includes temporary interruption or permanent discontinuation of linezolid. Percentages are calculated within each adverse event category. Patients may have experienced more than one hematological adverse event.
