# Supplementary material for: Prevalence and factors associated with hematological adverse events in RR-TB patients on linezolid-based regimens in Uganda: a multicenter retrospective cohort study
Source: BMC Infect Dis. 2026 Apr 30;26:1176. doi: 10.1186/s12879-026-13405-4 (PMC13289349; doi:10.1186/s12879-026-13405-4)
Supplement: Supplementary file 4 — Supplementary Material 4 [file 12879_2026_13405_MOESM4_ESM.pdf]

Supplementary Table S4. Management of Hematological Adverse Events by Adverse Event Type

Adverse event type	n	Permanent discontinuation n (%)	Temporary interruption or dose reduction n (%)	No linezolid modification n (%)
**Incident anemia**	51	5 (9.8)	1 (2.0)	45 (88.2)
**Incident leukopenia**	180	16 (8.9)	6 (3.3)	158 (87.8)

Note: Percentages are calculated within each adverse event category. Temporary interruption or dose reduction excludes patients who permanently discontinued linezolid. Patients may have experienced more than one hematological adverse event.
