# Supplementary material for: Prevalence and factors associated with hematological adverse events in RR-TB patients on linezolid-based regimens in Uganda: a multicenter retrospective cohort study
Source: BMC Infect Dis. 2026 Apr 30;26:1176. doi: 10.1186/s12879-026-13405-4 (PMC13289349; doi:10.1186/s12879-026-13405-4)
Supplement: Supplementary file 5 — Supplementary Material 5 [file 12879_2026_13405_MOESM5_ESM.pdf]

Supplementary Table S5: Sensitivity Analysis - Multivariable Poisson Regression for Incident Hematological Adverse Events in the Strictly Incident Cohort (n=81)

Characteristic	aPR	95% CI	p-value
**Residence**			
Rural	1.00 (Ref)		
Urban	1.00	0.92 – 1.09	0.979
**Marital Status**			
Single	0.80	0.53 – 1.22	0.306
Married	0.91	0.82 – 1.01	0.077
Widowed	1.04	0.91 – 1.18	0.585
**Age Group**			
≤20 years	1.00 (Ref)		
21 – 40 years	0.79	0.54 – 1.16	0.231
41 – 60 years	0.88	0.60 – 1.29	0.513
>60 years	0.67	0.40 – 1.11	0.117
**Sex**			
Female	1.00 (Ref)		
Male	1.06	0.93 – 1.20	0.382
**HIV Status**			
Negative	1.00 (Ref)		
Positive	1.04	0.96 – 1.13	0.286

**Diabetes History**			
No	1.00 (Ref)		
Yes	0.87	0.62 – 1.23	0.437

Abbreviations: aPR, adjusted prevalence ratio; CI, confidence interval.

Note: The strictly incident cohort included patients with normal baseline hemoglobin, white blood cell, and platelet counts (n=81). Cigarette smoking was excluded from this sensitivity analysis due to missing data (31 patients had missing smoking status). All models were adjusted for the covariates shown and used robust standard errors.
