# Supplementary material for: Prevalence and factors associated with hematological adverse events in RR-TB patients on linezolid-based regimens in Uganda: a multicenter retrospective cohort study
Source: BMC Infect Dis. 2026 Apr 30;26:1176. doi: 10.1186/s12879-026-13405-4 (PMC13289349; doi:10.1186/s12879-026-13405-4)
Supplement: Supplementary file 6 — Supplementary Material 6 [file 12879_2026_13405_MOESM6_ESM.pdf]

Supplementary Table S6. Multivariable analysis of factors associated with incident hematological adverse events restricted to HIV-negative patients (n=269).

Variable	aPR	95% CI	p-value
Rural residence (vs urban)	1.10	1.00–1.21	0.045
Married (vs single/never married)	0.98	0.87–1.09	0.687
Widowed/divorced (vs single/never married)	0.89	0.71–1.13	0.356
Cigarette smoking (yes vs no)	1.04	0.92–1.17	0.559
Age 21–40 (vs ≤20)	1.09	0.73–1.63	0.676
Age 41–60 (vs ≤20)	1.32	0.87–2.01	0.191
Age >60 (vs ≤20)	1.14	0.74–1.75	0.565
Male (vs female)	1.06	0.92–1.21	0.421
Diabetes (yes vs no)	0.06	0.03–0.13	<0.001

Note: Model adjusted for all variables shown. Modified Poisson regression with robust standard errors.
