# Supplementary material for: Prevalence and factors associated with hematological adverse events in RR-TB patients on linezolid-based regimens in Uganda: a multicenter retrospective cohort study
Source: BMC Infect Dis. 2026 Apr 30;26:1176. doi: 10.1186/s12879-026-13405-4 (PMC13289349; doi:10.1186/s12879-026-13405-4)
Supplement: Supplementary file 7 — Supplementary Material 7 [file 12879_2026_13405_MOESM7_ESM.pdf]

Supplementary Table S7. Multivariable analysis of factors associated with hematological adverse events using full model without p-value screening (n=311)

Variable	aPR	95% CI	p-value
**Residence**			
Urban	1.00 (Ref)		
Rural	1.34	1.09 – 1.64	0.005
**Nature of Employment**			
Employed	1.00 (Ref)		
Self-employed	1.13	0.84 – 1.52	0.411
Unemployed	0.75	0.56 – 1.02	0.071
**Marital Status**			
Single	1.00 (Ref)		
Married	0.66	0.53 – 0.84	0.001
Divorced/Widowed	0.91	0.63 – 1.31	0.624
**Cigarette Smoking**			
No	1.00 (Ref)		
Yes	1.67	1.35 – 2.07	<0.001
**HIV Status**			
Negative	1.00 (Ref)		
Positive	1.16	0.92 – 1.45	0.207
**Alcohol Use**			
No	1.00 (Ref)		

Yes	0.84	0.69 – 1.02	0.084
**Previous History of TB Treatment**			
No	1.00 (Ref)		
Yes	1.04	0.85 – 1.27	0.708

Note: Model adjusted for all a priori selected covariates shown. Modified Poisson regression with robust standard errors. Listwise deletion resulted in a sample size of 311 patients due to missing covariate data.
