# Supplementary material for: Prevalence and factors associated with hematological adverse events in RR-TB patients on linezolid-based regimens in Uganda: a multicenter retrospective cohort study
Source: BMC Infect Dis. 2026 Apr 30;26:1176. doi: 10.1186/s12879-026-13405-4 (PMC13289349; doi:10.1186/s12879-026-13405-4)
Supplement: Supplementary file 8 — Supplementary Material 8 [file 12879_2026_13405_MOESM8_ESM.pdf]

Supplementary Table S8: Baseline demographic and clinical characteristics by baseline CBC availability

Characteristic	Baseline CBC Available (n=245)	Baseline CBC Not Available (n=167)	P-value
**Age, mean (SD)**	41.3 (±16.96)	38.7 (±18.11)	0.128
**Residence, n (%)**			**0.021**
Rural	196 (80.0)	148 (88.6)	
Urban	49 (20.0)	19 (11.4)	
**Sex, n (%)**			0.181
Female	91 (37.1)	73 (43.7)	
Male	154 (62.9)	94 (56.3)	
**Marital Status, n (%)**			**0.007**
Divorced/Widowed	54 (22.0)	17 (10.2)	
Married	153 (62.4)	117 (70.1)	
Single	38 (15.5)	33 (19.8)	
**Cigarette Smoking, n (%)**			**<0.001**
No	56 (32.9)	89 (62.2)	
Yes	114 (67.1)	54 (37.8)	
**HIV Status, n (%)**			0.057
Negative	202 (82.4)	149 (89.2)	
Positive	43 (17.6)	18 (10.8)	

Note: For cigarette smoking, denominators are 170 (CBC available) and 143 (CBC not available) due to missing data.
