# Supplementary material for: Prevalence and factors associated with hematological adverse events in RR-TB patients on linezolid-based regimens in Uganda: a multicenter retrospective cohort study
Source: BMC Infect Dis. 2026 Apr 30;26:1176. doi: 10.1186/s12879-026-13405-4 (PMC13289349; doi:10.1186/s12879-026-13405-4)
Supplement: Supplementary file 9 — Supplementary Material 9 [file 12879_2026_13405_MOESM9_ESM.pdf]

Supplementary Table S9. Sensitivity analysis for surveillance bias – Poisson regression with adjustment for total CBCs after LZD initiation

Variable	IRR	95% CI	p-value
**Residence** (Urban vs Rural)	1.10	1.00 – 1.20	0.041
**Marital status** (Married vs Divorced/Widowed)	0.98	0.89 – 1.07	0.644
**Marital status** (Single vs Divorced/Widowed)	1.14	0.93 – 1.39	0.204
**Cigarette smoking** (Yes vs No)	0.98	0.88 – 1.09	0.678
**Age** (21–40 vs ≤20)	1.16	0.90 – 1.50	0.241
**Age** (41–60 vs ≤20)	1.42	1.07 – 1.88	0.015
**Age** (>60 vs ≤20)	1.26	0.95 – 1.68	0.114
**Sex** (Male vs Female)	1.07	0.95 – 1.19	0.257
**HIV status** (Positive vs Negative)	1.01	0.91 – 1.13	0.808
**Total CBCs after LZD initiation** (per additional CBC)	1.08	1.05 – 1.12	<0.001

Note: The variable for diabetes history showed an implausibly protective effect (IRR 0.10, 95% CI 0.05–0.21) and was excluded from the final model due to likely data coding issues or small numbers. The model presented includes all other covariates and was run on 313 patients with complete data.
